# Squeezing the arms as a treatment for acute ischemic stroke

**DOI:** 10.3389/fstro.2022.1039229

**Published:** 2022-10-24

**Authors:** Sean I. Savitz, Andrew D. Barreto

**Affiliations:** Institute for Stroke and Cerebrovascular Disease, Department of Neurology, University of Texas Health Science Center at Houston, Houston, TX, United States

**Keywords:** stroke, neuroprotection, recovery, ischemia, arm

In August, JAMA published (Chen et al., 2022) an open label randomized trial from China testing remote ischemic limb conditioning (RIC) in 1,893 patients with acute ischemic stroke. Supported by preclinical literature, the investigators aimed to test a new approach that they advocate is a neuroprotective strategy—inflate a cuff to both arms to cause limb ischemia. Preclinical studies have shown that remote limb ischemia reduces infarct size in rodent stroke models, and there have been smaller studies suggesting feasibility and safety of RIC in stroke patients. The study was conducted and published in a time frame when RIC is becoming very popular in the cardiac and rehabilitation literature.

Patients were randomized to usual care or usual care with the intervention. The intervention was 5 successive cycles of inflating a cuff placed around both arms to 200 mg Hg for 5 min followed by 5 min of deflation. The 5 cycles of compression were completed twice a day, started on average at 24 h after stroke, and were intended to continue for 14 days. Compared to the control group, there was a significantly higher percentage (5% difference) in the intervention group achieving an excellent outcome on the mRS disability score of 0–1.

Is RIC a new emerging treatment? In our opinion, the answer is no based solely on this publication. Here's a breakdown of our findings.

(1) **Significance and therapeutic strategy:** Why is this approach considered a neuroprotective strategy when it was applied on average 24 h after stroke? Classic neuroprotective strategies are intended to reduce infarct size and in modern times recommendations stress very early treatment and pairing with reperfusion. As the authors note, perhaps RIC is promoting recovery through unknown mechanisms?
(2) **Rigor:** Patients and physicians were not blinded. It is understandable that a sham procedure is difficult to develop but at least the application of the device on the arms should have been performed and perhaps cuff inflation to a nominal amount for a short duration. As a result, the outcome assessors could not have been blinded.
(3) **Sample size and treatment effect:** The estimated treatment effect of 7% was based on the results from the ECASS 3 trial (Hacke et al., 2008) but that study tested IV alteplase at 3–4.5 h after stroke onset—how would the treatment effect from that intervention be relevant to remote limb ischemia applied at 24 h after stroke? In addition, a small number of patients were randomized more than once. However, their data were analyzed appropriately.
(4) **Is this intervention relevant to our stroke population?** The patient population in this trial was highly selected. Patients were excluded if they received reperfusion therapy or had a cardioembolic stroke. Ultimately, the largest patient population turned out to be cryptogenic. The median NIHSS was 7 with IQR 6–9 in both groups. Thus, this was a patient population with fairly low severity. The study does not provide meaningful data on NIHSS > 10. And, the entire duration of the intervention occurred in the hospital. We need a “take-home” device for widespread application of RIC. Alternatively, subsequent trials should consider testing an abbreviated treatment algorithm.
(5) **Variation in treatment:** Only 6% of the study patients actually completed the full 14 days of treatment. The reasons are not fully explained. The number of days that patients underwent the intervention was quite variable anywhere from < 8 days to 14 days because many patients dropped out of the study. However, this issue presents an opportunity to determine if there was a dose-response relationship. Is there an association between duration of treatment and outcome? That question was not tested in this trial but was there even a signal of increasing response (% of mRS 0–1) in patients with higher number of days of RIC treatment? Since not all RIC patients received the full treatment duration, an exploratory analysis testing number of sessions with outcome would be interesting. Could there be a gradient effect as included in the Bradford-Hill causation criteria?
(6) **Analyses and confounders:** We found it puzzling that the investigators excluded after randomization a non-trivial number of patients. These patients should have been part of intention to treat analyses. There was also no specified analysis to test for a center effect. Could the results have been influenced based on where the study took place in individual hospitals? Maybe some hospitals perform better in implementation of guideline-endorsed standard of care. In addition, the specific medications and amount of rehabilitation that each patient received was not provided.
(7) **Other interesting issues:** There was significant drop out of patients but only 6 due to the intolerance of the procedure. Surprisingly, pain was listed to have occurred in zero patients. We all understand the uncomfortable feeling of blood pressure testing (especially to 200 mg Hg) and 5 min would be a long time for many patients. What effects does arm compression have on patients with concomitant peripheral arterial disease which undoubtedly many of the patients must have had, given high percentages of vascular comorbidities?

In summary, we think the trial is thought provoking. Is RIC worth further study to promote recovery after stroke? Inflating a cuff to the arms is easy, should not be costly, and likely poses some discomfort that many will consider worth tolerating to achieve better recovery after stroke. We hope to see a replication trial that takes into account the above issues in a diverse patient population and analyzes all data from randomized patients. We would recommend a multi-arm trial that includes a better control and can test different types/duration of the RIC. Adding an arm that only undergoes treatment for a set number of days, that reflects the shorter length of stay of other countries, should be considered. Lastly, we recommend a secondary outcome assessment using a standardized quality of life measurement (e.g., EQ-5D-5L) which can provide additional evidence of a treatment effect.

## Author contributions

Both authors listed have made a substantial, direct, and intellectual contribution to the work and approved it for publication.

## Conflict of interest

The authors declare that the research was conducted in the absence of any commercial or financial relationships that could be construed as a potential conflict of interest.

## Publisher's note

All claims expressed in this article are solely those of the authors and do not necessarily represent those of their affiliated organizations, or those of the publisher, the editors and the reviewers. Any product that may be evaluated in this article, or claim that may be made by its manufacturer, is not guaranteed or endorsed by the publisher.
